# Cardiac disease is linked to adiposity in male gorillas (*Gorilla gorilla gorilla*)

**DOI:** 10.1371/journal.pone.0218763

**Published:** 2019-06-26

**Authors:** Patricia M. Dennis, Mary Ann Raghanti, Richard S. Meindl, Elena Less, Eric Henthorn, William Devlin, Suzan Murray, Thomas Meehan, Ilana Kutinsky, Hayley Murphy

**Affiliations:** 1 Cleveland Metroparks Zoo, Cleveland, Ohio, United States of America; 2 Department of Veterinary Preventive Medicine, The Ohio State University, Columbus, Ohio, United States of America; 3 Department of Anthropology and School of Biomedical Sciences, Kent State University, Kent, Ohio, United States of America; 4 Brain Health Research Institute, Kent State University, Kent, Ohio, United States of America; 5 Fortis College Cuyahoga Falls, Ohio, United States of America; 6 Oakland University William Beaumont School of Medicine, Rochester, Michigan, United States of America; 7 Beaumont Michigan Heart Group, Troy, Michigan, United States of America; 8 Smithsonian Conservation Biology Institute, National Zoological Park, Washington DC, United States of America; 9 Brookfield Zoo, Brookfield, Illinois, United States of America; 10 Great Ape Heart Project based at Zoo Atlanta, Zoo Atlanta, Atlanta, Georgia, United States of America; Stellenbosch University, SOUTH AFRICA

## Abstract

Cardiac disease is a major cause of morbidity and mortality for adult gorillas. Previous research indicates a sex-based difference with predominantly males demonstrating evidence of left ventricular hypertrophy. To evaluate these findings, we analyzed serum markers with cardiac measures in a large sample of gorillas. The study sample included 44 male and 25 female gorillas housed at American Association of Zoo and Aquariums (AZA)-accredited zoos. Serum samples were collected from fasted gorillas during routine veterinary health exams and analyzed to measure leptin, adiponectin, IGF-1, insulin, ferritin, glucose, triglycerides, and cholesterol. Cardiac ultrasonography via transthoracic echocardiogram was performed simultaneously. Three echocardiographic parameters were chosen to assess cardiac disease according to parameters established for captive lowland gorillas: left ventricular internal diameter, inter-ventricular septum thickness, and left ventricular posterior wall thickness. Our data revealed that high leptin, low adiponectin, and lowered cholesterol were significantly and positively correlated with measures of heart thickness and age in males but not in females. Lowered cholesterol in this population would be categorized as elevated in humans. High leptin and low adiponectin are indicative of increased adiposity and suggests a potential parallel with human obesity and cardiovascular disease in males. Interestingly, while females exhibited increased adiposity with age, they did not progress to cardiac disease.

## Introduction

Cardiac disease is a major cause of death for gorillas in zoological settings [1, 2]. This is a male-biased disease, characterized by left ventricular hypertrophy and cardiomyopathy, but with an absence of atherosclerosis [1, 3]. In humans, left ventricular dysfunction and clinical heart failure are associated with obesity and insulin resistance [4, 5]. Previous studies demonstrated that both obesity and insulin resistance occur in captive gorillas [6], raising the possibility that these factors are associated with gorilla heart disease. Understanding the epidemiology and risk factors of heart disease in gorillas is critical for managing their health in zoo settings, as their status in the wild in critically endangered [7].

## Materials and methods

The study sample included 44 male (ages 4–50 years, mean 21.4 ± 10.99) and 25 female (ages 5 to 51 years, mean 21.3 ± 7.35) gorillas housed at AZA-accredited zoos. This research was reviewed and approved by Cleveland Metroparks Zoo Scientific Review Committee. Serum samples were collected from fasted gorillas during routine veterinary health exams by or under the supervision of a veterinarian. Serum samples were analyzed using enzyme immunoassay using commercially available kits for leptin (Mercodia 10-1199-01), adiponectin (B-Bridge K-1002-1), IGF-1 (IDS AC-27), insulin (Mercodia 10-1132-01), and ferritin (Calbiotech FR065T). Each of these assays was validated for use in gorillas by the Cleveland Metroparks Zoo Endocrinology Laboratory. For each assay, a pooled sample dilution curve was parallel to the standard curve with no difference between slopes (adiponectin t_(10)_ = 1.03, p > 0.05; leptin t_(9)_ = 0.1, p > 0.05; insulin t_(9)_ = 1.43, p > 0.05; IGF-1 t_(15)_ = 0.37, p > 0.05; ferritin t_(10)_ = 0.58, p > 0.05). Recovery for high and low concentrations was > 90% for each concentration and assay. Glucose, triglycerides, and cholesterol were measured using an IDEXX Vettest 8008 Chemistry Autoanalyzer.

Cardiac ultrasonography via transthoracic echocardiogram was performed at the same time using standard protocols [1]. Three echocardiographic parameters were chosen to assess cardiac disease according to parameters established for captive lowland gorillas [1]. These measures were chosen because progressive left ventricular hypertrophy is the predominant finding in heart disease of male gorillas. These were left ventricular internal diameter (LVID), inter-ventricular septum thickness (IVS), and the left ventricular posterior wall thickness (LVPW).

## Statistical analyses

For the male and female samples separately, Spearman’s partial correlation coefficients, i.e., controlling for age, were calculated among the six hematology markers as well as the glucose-to-insulin ratio. Using Spearman’s partial correlation coefficients normalized the outliers that were present within the sample. Principal components analyses with varimax rotation were then performed on each Spearman matrix to correct for general skewness and outliers. The major principal components were then analyzed for relationships with echocardiograph measures indicative of heart disease in make gorillas using Pearson’s r correlation analyses.

## Results

### Levels of blood markers and heart thicknesses, by sex

#### Males

Summary statistics for all measures are provided in Table 1 and raw data are provided in S1 Table. Principal component (PC) 1 explained 26% of the variation (Table 2). High values of PC1 corresponded to high values of leptin, and low values of both adiponectin and cholesterol. High leptin with low adiponectin is associated with increased adiposity in captive gorillas [8]. Within this sample, mean cholesterol was higher than 200 mg/dl, which is not unusual for this species but higher than what is typically observed in humans. A mean of 284 mg/dl was previously reported for captive gorillas, which was 1.5 times higher than for free-ranging gorillas [9]. Taken together, PC1 is most indicative of elevated adiposity and cholesterol, although decreased relative to the present sample, would still be elevated compared with wild gorilla or normal human populations.

**Table 1:** Summary data for serum biomarkers and heart measures.

	Males (N = 44)	Females (N = 25)
**Age (years)**	21.4 ± 10.99	21.3 ± 7.35
**Serum biomarkers**		
Leptin (ng/mL)	8.51 ± 10.07	13.25 ± 9.03
Adiponectin (ng/mL)	0.89 ± 0.90	0.82 ± 0.79
IGF-1 (μg/L)	369.66 ± 165.59	202.02 ± 99.03
Insulin (mU/L)	8.78 ± 10.29	8.13 ± 16.33
Glucose (mg/dL)	89.16 ± 15.39	101.32 ± 33.17
Glucose:Insulin ratio	43.46 ± 53.91	52.87 ± 56.61
Ferritin (ng/mL)	431.56 ± 208.85	667.09 ± 976.74
Cholesterol (mg/dL)	255.45 ± 63.64	217.88 ± 66.03
Triglycerides (mg/dL)	127.05 ± 64.01	162.08 ± 84.31
**Heart measurements**		
LVID (mm)	5.89 ± 1.06	4.34 ± 0.66
IVS (mm)	1.47 ± 0.43	1.12 ± 0.30
LVPW (mm)	1.48 ± 0.40	1.16 ± 0.31

**Table 2:** Principal component factor loadings (varimax normalized).

	Factor 1		Factor 2		Factor 3	
	Males	Females	Males	Females	Males	Females
Leptin	**0.67**	**0.79**	0.08	0.19	0.57	0.15
Cholesterol	**-0.80**	**-0.75**	-0.09	0.47	-0.02	-0.11
Adiponectin	**-0.68**	**-0.67**	0.02	-0.43	-0.06	0.28
IGF-1	0.16	0.03	**0.86**	**0.83**	-0.20	0.33
Ferritin	0.14	0.13	**-0.62**	-0.21	-0.53	**-0.89**
Triglycerides	0.47	0.21	-0.05	-0.16	**0.71**	**0.72**
Glucose:Insulin	0.01	-0.10	0.07	**-0.70**	**-0.79**	0.15
**% Variation Explained**	**26%**	**25%**	**16%**	**24%**	**25%**	**22%**

PC2 explained 16% of total blood marker variation, with high values of PC2 reflecting high IGF1 (see Table 2). PC3 explained 25% of the variation. High values of PC3 reflect high triglycerides and a low glucose-to-insulin ratio (See Table 2). Taken together, both PC2 and PC3 are suggestive of dysregulated glucose metabolism as a major component explaining the variation within serum biomarkers in the males.

**Fig 1:**
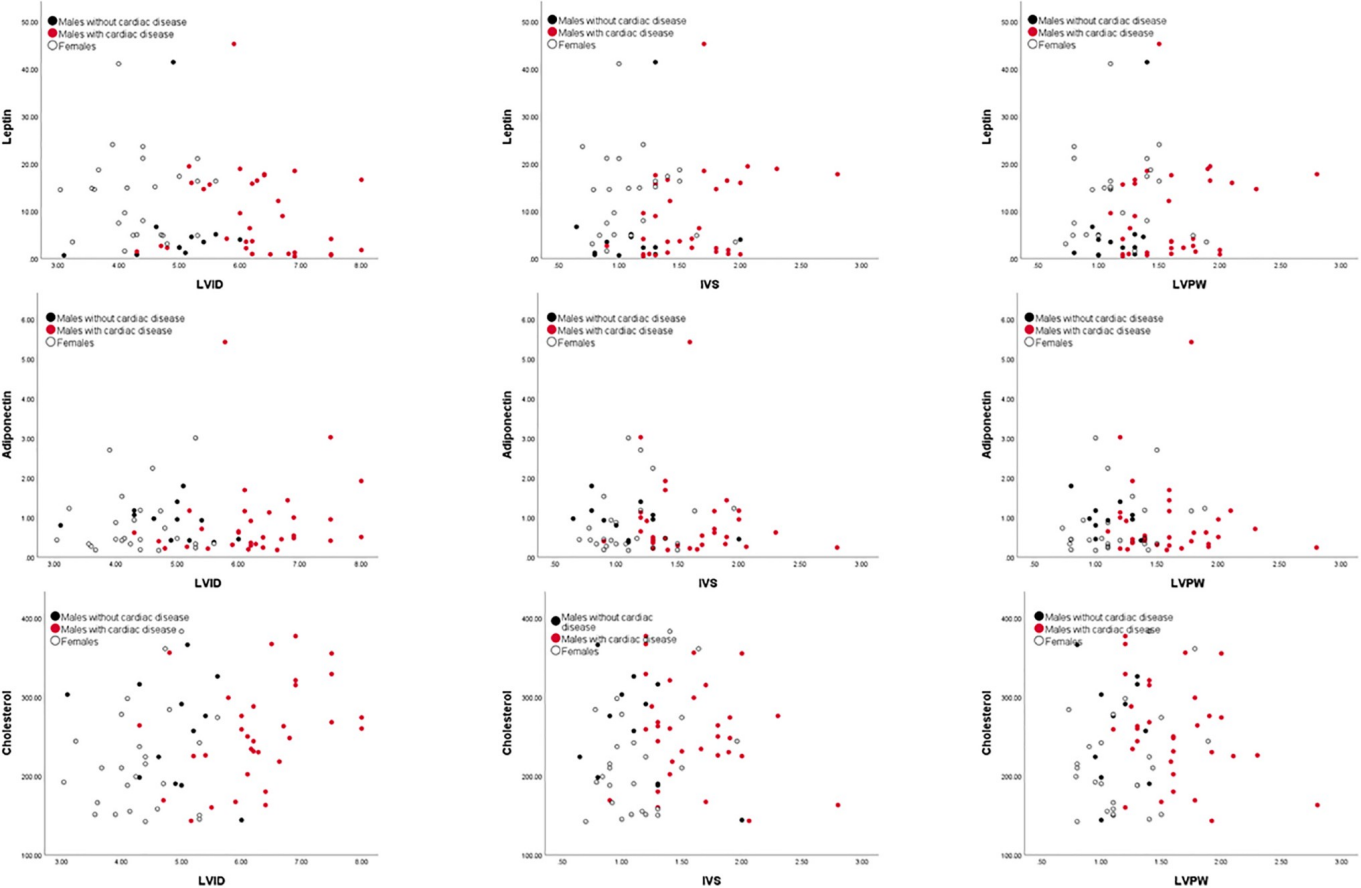
The significant variables for principal component 1 plotted against heart measures. Leptin, adiponectin and cholesterol are each plotted against heart measures LVID, IVS, and LVPW. For males, principal component 1 was significantly correlated with heart measures IVS and LVPW. Males that were previously diagnosed with heart disease are indicated by red circles, healthy males are depicted by black circles and females by white circles.

**Fig 2:**
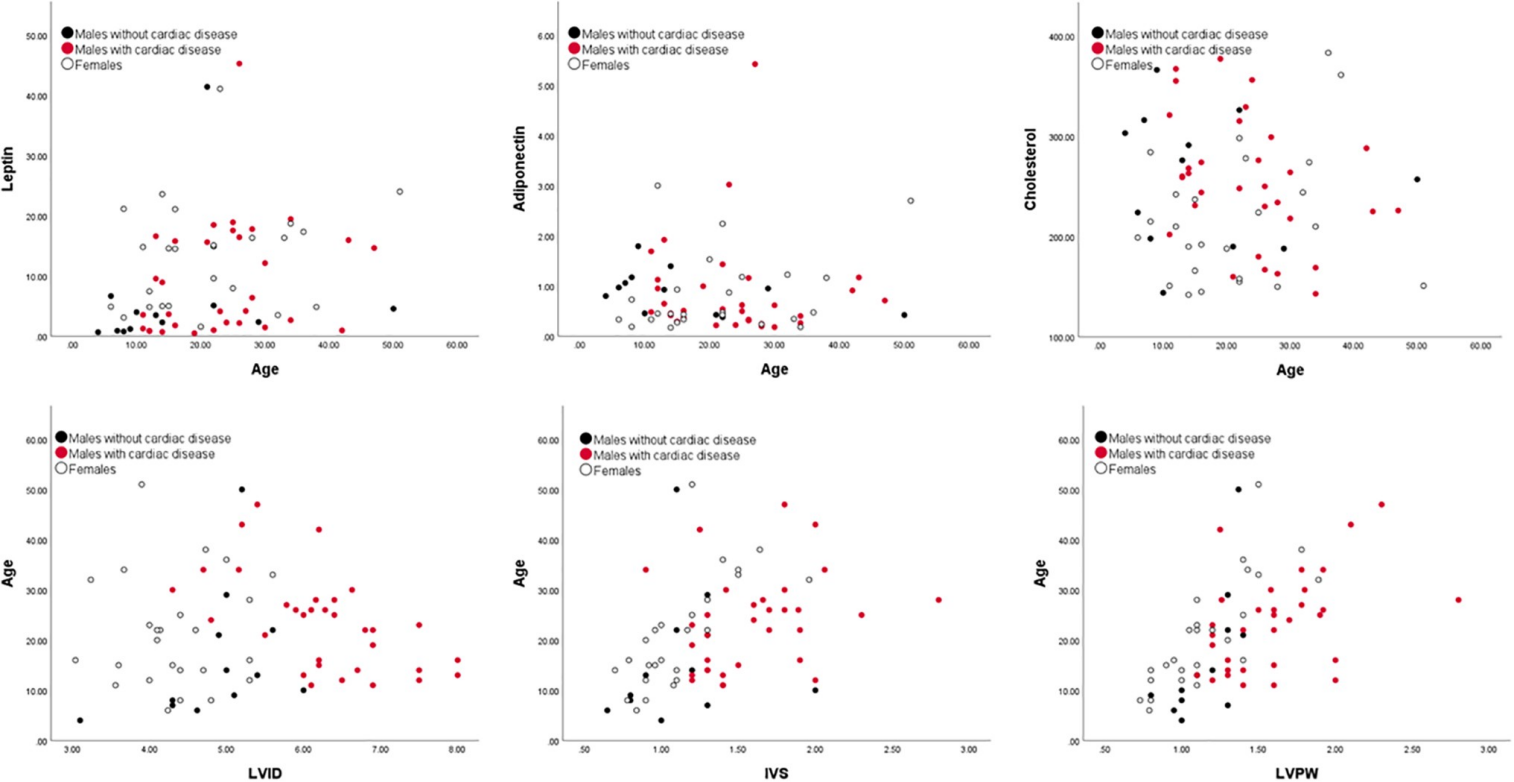
The significant variables for principal component 1 plotted against age and age plotted against heart measures. Leptin, adiponectin and cholesterol are each plotted against age. For males, principal component 1 was significantly correlated with age. Age is also plotted against each heart measure. Males that were previously diagnosed with heart disease are indicated by red circles, healthy males are depicted by black circles and females by white circles.

Principal components were then compared with the echocardiogram measures (Table 3, Figs 1 and 2). In males, PC1 increased with the two heart measures of IVS (Pearson’s r = 0.39, p < 0.01) and LVPW (Pearson’s r = 0.40, p < 0.01) and with age (Pearson’s r = 0.42, p < 0.01; see Table 3, Figs 1 and 2). PC2 correlated positively with age (Pearson’s r = 0.69, p < 0.01), but not with any of the heart measures (all p’s > 0.05). PC3 did not correlate with age or any heart measure (all p’s > 0.05).

**Table 3:** Pearson correlations of principal components factor loadings with heart measures and age. The first number is the Pearson correlation coefficient, the second is the significance level (2-tailed).

	LVID	IVS	LVPW	Age
**Males Factor 1**	0.01, p = 0.94	**0.39, p < 0.01**	**0.40, p < 0.01**	**0.42, p < 0.01**
**Males Factor 2**	0.14, p = 0.36	0.06, p = 0.70	0.20, p = 0.21	**0.69, p < 0.01**
**Males Factor 3**	0.22, p = 0.16	0.03, p = 0.87	0.04, p = 0.79	-0.14, p = 0.38
**Females Factor 1**	0.20, p = 0.33	0.33, p = 0.11	0.28, p = 0.18	0.20, p = 0.35
**Females Factor 2**	0.14, p = 0.50	0.14, p = 0.51	0.11, p = 0.60	0.18, p = 0.35
**Females Factor 3**	-0.07, p = 0.74	0.15, p = 0.48	0.17, p = 0.41	-0.03, p = 0.88

This multivariate approach appears to identify the leptin/adiponectin ratio coupled with the inverse of cholesterol as a major axis of variation in the blood marker system as well as a primary correlate of heart thickness in male gorillas.

#### Females

For the sample of 25 adult female gorillas the same multivariate axes were examined. While the sample of females was smaller, the eigen-solutions for the male and female samples were remarkably consistent, which implies that the blood marker inter-correlation structures were similar. Summary statistics can be found in Table 1, raw data are provided in S1 Table.

PC1 explained 25% of the female gorilla blood marker variation. As in males, the high values of PC1 reflected high leptin, low adiponectin, and lowered cholesterol (Table 2). The PC1 pairs of numerical loadings were virtually the same, i.e., the first eigenvectors for males and females represent equivalent principal components. PC2 represented 24% of the variation, and was strongly positively correlated with high IGF1, as was the case of the males. The difference in the correlation structure comparing males and females was that in PC2 the only other large loading for females was the glucose-to-insulin ratio vs. ferritin in males. PC3 loaded the same as males for triglycerides. The only other difference between the sexes was the second highest loading for PC3 was low glucose-to-insulin ratio in males versus low ferritin for females (Table 2, Figs 1 and 2).

While the bivariate and multivariate correlation and principal component structures were very similar in both sexes, there was no significant correlation between the measures of heart disease or age in females (Table 3). Although the age distributions of both males and females were very similar, the heart thicknesses were significantly smaller in females, (Figs 1 and 2); therefore, females simply have not progressed as far as males out on the first component, despite equivalent ages. That is, the primary dimensions of risk of heart disease are similar in both sexes, however for all practical purposes the older females appear to have the cardiac thicknesses of younger males.

## Discussion

The present results indicate a strong relationship between adiposity and heart disease in captive male gorillas. Interestingly, females show a similar relationship between age and obesity, yet no association between adiposity and heart disease. In terms of body composition, adiposity refers to the amount of fat an organism possesses [10]. Obesity, it turn, is defined as having excess adipose tissue relative to lean body mass [11]. Accurate measures of body condition account for both body weight and lean mass (i.e., body mass index, BMI), but these measures may not always be easily transferred to nonhuman species. As hindgut fermenters, gorillas possess an extensive colon for processing fiber, making typical measures, such as waist circumference, less useful. Our previous work showed that, for male gorillas, absolute weight was the best predictor of the serum biomarkers of leptin and adiponectin [6, 8]. In females, leptin was positively correlated with hip width, shoulder width, and widest point [8]. Taken together, leptin is the best predictor of body condition in captive gorillas.

In humans, high leptin is associated with cardiac hypertrophy and obesity [12]. Adiponectin protects cardiomyocytes from hypertrophy [13]. We found similar patterns in male gorillas, with high leptin and low adiponectin associated with indicators of left ventricular hypertrophy. While the same patterns of biomarkers were present in female gorillas, there was no association with left ventricular hypertrophy. With left ventricular hypertrophy in humans, independent of blood pressure and other cardiovascular risk factors, women are less prone to LVH than men until women enter menopause [14]. Estrogens attenuate cardiac hypertrophy in mouse model by downregulating prohypertrophic signaling pathways that lead to cardiac hypertrophy [15]. Estrogens may serve to prevent cardiac hypertrophy in female gorillas exhibiting similar conditions to those of males. Testosterone does not provide a cardioprotective mechanism, rather, has been demonstrated to induce cardiac myocyte hypertrophy [16]. In human males, obesity and aging are associated with lower serum testosterone levels [17]. This suggests that high testosterone levels may not be a factor in left ventricular hypertrophy in male gorillas, as it is associated with age and obesity.

The finding of an association between heart disease and lowered serum cholesterol in males is surprising and seemingly in contrast to what is known regarding heart disease in humans. Studies in human health identify hyperlipidemia as a risk factor in cardiovascular disease [18]. Other studies have examined the influence of the gut microbiota on cholesterol metabolism [19]. The gut microbiota are also shown to have significant effect on cardiovascular health [20]. We previously demonstrated that the fecal microbiome of male gorillas with cardiac disease differs from that of healthy males [21]. A future avenue of investigation will focus on the role of the gorilla gut microbiome in cholesterol metabolism in association with heart disease.

## Supporting information

S1 Table Raw data. All data used for the present analyses are included here. For the column labeled sex, F = female, M = male. Age is provide in years. Units for serum markers and heart measures are provided in Table 1 of the main text.
(XLSX)
